# Editorial: Advancing Health Technology Assessment and patient-reported outcomes: innovations and implications for health economics and outcomes research

**DOI:** 10.3389/fpubh.2026.1898322

**Published:** 2026-06-25

**Authors:** George Gourzoulidis, Catherine Kastanioti, George Mavridoglou, Theodore Kotsilieris, Charalampos Tzanetakos

**Affiliations:** 1 Health Through Evidence, Athens, Greece; 2 Department of Business and Organizations Administration, University of the Peloponnese, Kalamata, Greece; 3 Department of Accounting and Finance, University of the Peloponnese, Kalamata, Greece

**Keywords:** Health Technology Assessment (HTA), outcomes research, patient-centered care, patient-reported outcome measures (PROM), patient-reported outcomes (PRO)

Health Technology Assessment (HTA) has become a central instrument for evidence-informed healthcare decision-making, supporting reimbursement, pricing, prioritization, and resource allocation across health systems facing increasing clinical, economic, and societal pressure. The internationally accepted definition of HTA describes it as a multidisciplinary process that uses explicit methods to determine the value of health technologies at different points in their lifecycle, with the purpose of promoting equitable, efficient, and high-quality health systems (1). This definition is especially relevant today, as precision medicines, immunotherapies, cell and gene therapies, diagnostics, digital health interventions, and preventive technologies create new opportunities for patients while intensifying questions about affordability, access, uncertainty, and long-term value (2).

Traditional HTA frameworks have relied primarily on clinical endpoints, safety outcomes, cost-effectiveness analyses, and budget impact assessments. These dimensions remain indispensable, but they do not always capture the full impact of health technologies on everyday life. Symptom burden, physical functioning, emotional wellbeing, autonomy, treatment convenience, caregiver impact, social participation, and patient preferences may be highly relevant to patients even when they are only partially reflected in conventional endpoints (3). Technology may improve a clinical outcome while imposing substantial treatment burden; conversely, an intervention with modest clinical effects may produce meaningful gains in reassurance, adherence, functioning, or quality of life (4). Contemporary HTA is therefore evolving from a predominantly technical assessment of effectiveness and efficiency into a more multidimensional and deliberative process.

Patient-reported outcomes (PROs), patient-reported outcome measures (PROMs), patient experience, and meaningful patient involvement are central to this evolution. Patient-centered HTA should not be reduced to the mechanical inclusion of questionnaires or utility values (5). Rather, it requires both robust evidence about patient perspectives and effective engagement of patients in scoping, evidence generation, value assessment, recommendation development, and dissemination (6). Recent patient-reported surveys illustrate why this matters: chronic disease burden may remain substantial despite advanced therapy use, affecting quality of life, work productivity, mental health, treatment satisfaction, and adherence (7, 8). Such evidence highlights that the value of care cannot be inferred from biomedical markers alone.

PROs and PROMs also create a bridge between clinical research and economic evaluation. Preference-based measures can inform utility estimation, while disease-specific instruments and qualitative patient evidence can explain what changes are meaningful, acceptable, or burdensome from the patient perspective (9). Their contribution is greatest when they are planned early, collected consistently, interpreted in context, and connected to decisions about comparators, endpoints, model assumptions, and reimbursement conditions. In this sense, patient-centered evidence should be understood not as an optional supplement to HTA, but as part of the evidence infrastructure required to assess value responsibly (10).

Meaningful patient involvement is broader than consultation at the final stage of appraisal. Patients and caregivers can help define the decision problem, identify relevant comparators, guide endpoint selection, interpret trade-offs between benefits and harms, and clarify which uncertainties are acceptable in practice (10). They can also reveal aspects of value that may otherwise remain invisible, such as travel time, monitoring burden, stigma, family disruption, financial vulnerability, and the practical usability of care (11). Embedding these perspectives early can improve scientific relevance and deliberative legitimacy, reducing the risk that HTA processes answer questions that are technically precise but socially incomplete.

At the same time, health systems must reconcile patient-centered value with fiscal sustainability. This is particularly evident in oncology and precision medicine, where targeted therapies and biomarker-driven treatment pathways have transformed care but also increased pressure on reimbursement systems (2, 12). This evidence reinforces the need for HTA frameworks that integrate clinical benefit, patient value, real-world utilization, economic impact, and affordability.

The present Research Topic, *Advancing Health Technology Assessment and patient-reported outcomes: innovations and implications for health economics and outcomes research*, brings together 19 contributions that reflect this transformation across diseases, technologies, methods, and health-system settings. The Research Topic is not simply a set of disease-specific evaluations. It illustrates a broader agenda for HTA and Health Economics and Outcomes Research (HEOR): how to generate and interpret evidence that is clinically meaningful, economically robust, methodologically transparent, and aligned with patient and societal priorities.

A first group of studies foregrounds burden, quality of life, and unmet need. The studies on severe hemophilia in Greece (Sofiaki et al.), gout and hyperuricemia (Zheng et al.), homozygous familial hypercholesterolemia (Tang, Ye et al.), and respiratory syncytial virus disease and maternal immunization (Gourzoulidis et al.) collectively show that disease burden is not limited to clinical events or laboratory indicators. It is experienced through symptoms, functional limitations, anxiety, family responsibilities, time costs, access barriers, and financial consequences. For HTA, these dimensions are not peripheral. When lived burden is insufficiently measured, the value of an intervention may be underestimated; when patient priorities are not reflected in endpoints or models, assessment may fail to address outcomes that matter most.

A second group highlights patient engagement, communication, and healthcare experience. The randomized trial of WeChat-based patient-doctor interaction (Lin et al.) for Helicobacter pylori treatment, the randomized study of motion-graphic video for informed consent (Mettarikanon et al.) in platelet-rich plasma therapy, and the qualitative study on medical device incident reporting (Mishali et al.) demonstrate that patient-centered value depends not only on the technology itself, but also on how care is communicated, understood, monitored, and governed. These studies broaden the meaning of patient-centered HTA beyond PROMs alone, emphasizing trust, participation, safety, autonomy, and responsiveness. They also remind us that implementation processes can shape the real-world value of a technology as much as its intrinsic clinical properties.

Economic evaluation represents another central theme. The analyses of thrombopoietin receptor agonists for chronic immune thrombocytopenia (Jin et al.), PCSK9 inhibitors for hypercholesterolemia (Hu et al.), semaglutide vs. liraglutide for obesity in Greece (Papantoniou et al.), prosthodontic rehabilitation in Greece (Chatzidou et al.), and disease-modifying therapies for relapsing-remitting multiple sclerosis in Saudi Arabia (Al-Jedai et al.) show how model structure, comparator selection, local prices, epidemiology, treatment pathways, and budget impact influence interpretation of value. The oncology studies of sugemalimab plus chemotherapy for advanced gastric or gastroesophageal junction cancer (Zhang, He et al.), sugemalimab combined with chemotherapy for advanced gastric cancer (Tang, Zhu et al.), and cadonilimab plus chemotherapy for HER2-negative advanced gastric cancer (Zhang, Yang et al.) further illustrate the pressure created by high-cost innovation. Together, these studies show that economic evaluation is not merely a technical calculation of incremental cost-effectiveness ratios. It is a policy tool for examining opportunity costs, affordability, uncertainty, and the conditions under which access to innovation can be justified.

Several contributions also address methodological and institutional adaptation. The analysis of HTA outcomes for cell and gene therapies (Almalki et al.) highlights the difficulty of assessing technologies with high upfront costs, limited long-term data, small patient populations, and potentially transformative benefits. The perspective on economic modeling in diagnostic test accuracy studies (Xie et al.) shows the need to connect accuracy with downstream pathways, patient outcomes, costs, and quality of life. The study on augmented reality in health media campaigns (Habes et al.) expands the scope of evaluation to behavioral and digital interventions, where engagement, implementation, scalability, and public health impact are central. Finally, the study on strengthening HTA in Greece (George et al.) emphasizes that credible assessment depends not only on methods, but also on capacity, transparency, stakeholder engagement, consistency, and timely decision-making.

Across these contributions, a coherent message emerges modern HTA must remain analytically rigorous while becoming more inclusive and socially responsive. Clinical effectiveness and economic efficiency remain fundamental, but they are not sufficient as stand-alone indicators of value. Healthcare includes survival, symptom control, safety, quality of life, convenience, autonomy, equity, affordability, implementation feasibility, and trust. Capturing these dimensions requires broader evidence, stronger methodological integration, and more deliberate involvement of patients, caregivers, clinicians, payers, industry, policymakers, and society (13).

This also requires careful attention to context. Costs, thresholds, clinical pathways, epidemiology, reimbursement rules, implementation capacity, and societal preferences differ substantially across healthcare systems. Evidence generated in one setting may be informative, but it cannot always be transferred without adaptation. Conversely, local analyses are most useful when they follow transparent methods and allow decision-makers to understand uncertainty, assumptions, and generalizability. The balance between methodological consistency and local relevance is therefore central to the development of credible and internationally useful HTA.

Uncertainty is another cross-cutting issue. For advanced therapies, rare diseases, diagnostics, digital interventions, and prevention strategies, evidence at launch may be immature, indirect, or incomplete. HTA systems may therefore require conditional reimbursement, managed entry agreements, registries, post-adoption evidence generation, and reassessment. Patient involvement is especially important under uncertainty, because patients may differ in their willingness to accept risk, tolerate evidence gaps, or value future benefits. Transparent deliberation can make these trade-offs explicit and support decisions that are both evidence-informed and socially defensible (14, 15).

Looking forward, the future of patient-centered HTA will depend on systematic patient involvement across the full assessment lifecycle: topic prioritization, study design, endpoint selection, evidence generation, appraisal, deliberation, implementation, and reassessment. Real-world evidence and patient-generated health data can help reduce uncertainty after adoption, particularly for advanced therapies, rare diseases, diagnostics, digital tools, and preventive interventions. However, the goal should not simply be to collect more patient data. PROMs, patient experience measures, and qualitative evidence should be selected, interpreted, and applied in ways that influence the questions HTA asks and the decisions it supports.

This agenda also has an important equity dimension. Patient involvement can reveal differences in access, affordability, health literacy, geography, cultural acceptability, and treatment burden that may remain invisible in aggregate models. For decision-makers, incorporating these perspectives can improve the fairness and legitimacy of priority-setting when resources are constrained. For researchers, it creates a responsibility to design studies and models that are not only technically valid, but also responsive to the populations whose outcomes they are intended to improve.

For HEOR researchers, these developments call for closer integration between clinical studies, qualitative research, preference studies, economic modeling, and implementation science. Trial protocols and observational studies should anticipate the evidence needs of HTA bodies, while models should make explicit how patient-reported outcomes, patient preferences, caregiver effects, and equity considerations are included or excluded. For HTA agencies, patient evidence should be submitted, appraised, discussed, and reported transparently. Patient involvement should not be treated as a parallel activity separated from technical assessment, but as part of the evidentiary and deliberative architecture through which health-system choices are made.

In conclusion, the articles in this Research Topic collectively demonstrate the growing importance of integrating patient-centered evidence, economic evaluation, methodological innovation, and stakeholder participation within modern healthcare decision-making. As health systems confront increasing complexity, uncertainty, and resource constraints, HTA must continue evolving toward frameworks that are transparent, adaptive, inclusive, and responsive to societal values. Ultimately, the future of HTA will depend not only on methodological rigor, but also on the willingness of health systems to meaningfully incorporate the experiences, priorities, and values of the patients they are intended to serve.
